# Biological Activity of Quaternary Ammonium Salts and Their Derivatives

**DOI:** 10.3390/pathogens9060459

**Published:** 2020-06-10

**Authors:** Dobrawa Kwaśniewska, Ying-Lien Chen, Daria Wieczorek

**Affiliations:** 1 Department of Technology and Instrumental Analysis, Institute of Quality Science, Poznań University of Economics and Business, 61-875 Poznań, Poland; dobrawa.kwasniewska@ue.poznan.pl; 2 Department of Plant Pathology and Microbiology, National Taiwan University, Taipei 100, Taiwan; ychen28@ntu.edu.tw

**Keywords:** quaternary ammonium salts, sulfobetaines, biocidal activity

## Abstract

Besides their positive role, microorganisms are related to a number of undesirable effects, including many diseases, biodeterioration and food spoilage, so when their presence is undesired, they must be controlled. Numerous biocides limiting the development of microorganisms have been proposed, however, in this paper the biocidal and inhibitory activity of quaternary ammonium salts (QASs) and their zwitterionic derivatives is addressed. This paper presents the current state of knowledge about the biocidal activity of QAS and their derivatives. Moreover, the known mechanisms of antimicrobial activity and the problem of emerging resistance to QAS are discussed. The latest trends in the study of surfactants and their potential use are also presented.

## 1. Mechanism of Biocidal Action

People have been fighting microbes for centuries, although at first, they were not aware what was the source of the problems. The shelf life of water was extended by storing it in silver vessels or by inserting coins made of silver or copper. Food was preserved using salt or spices. Later, phenol was used in hospitals as a disinfectant, but the real breakthrough in the fight against bacteria was the discovery of penicillin in the late 1920s. Its application has brought a significant decrease in mortality and morbidity among humans and animals. Currently, infection control, apart from the use of antibiotics, also includes the extensive use of biocides [1,2]. Antibiotics are used as anti-infective agents in the body’s tissues due to its high antimicrobial potency and selective toxicity. They usually have a highly specific effect; at therapeutic concentrations they inhibit growth and interact with the immune system. Such interaction with the immune system is not observed in the case of biocides [3]. The main characteristic of antibiotics is their origin: it is estimated that more than 5000 different antibiotics have been isolated from cultures of bacteria, fungi and plant cells [4]. The development of biotechnology and genetics meant that the majority of clinically used antibiotics are derived from bacterial small molecules produced by dedicated biosynthetic gene clusters [5] The term "biocides" defines a group of chemical compounds whose main task is to inactivate or kill vegetative microorganisms. Due to their biocidal potential, they are used for disinfectant, antiseptic, sterilant, preservative treatments. Chemical compounds that are used on the skin or living tissue to kill or inhibit the growth of vegetative microorganisms are treated as antiseptic products. To obtain microbiologically clean inanimate surfaces, disinfectant products are used, and the primary purpose of these biocides is to kill or inactivate the vegetative form of bacteria. In medical applications, where high microbiological purity is expected, sterilant compositions are mainly used. Their role is to kill vegetative and spore-forming bacteria. The last group of biocides mainly used to prevent the development of organisms, resulting in the deterioration of the quality of the product, are preservatives [6]. The biocidal activity of a biocide depends, however, not only on its chemical structure, but also on the type of pathogen it is supposed to fight. In simple terms, it was established that the most resistant to disinfection are the mycobacteria, while Gram-negative bacteria and Gram-positive bacteria are the most sensitive [7].

**Figure 1:**
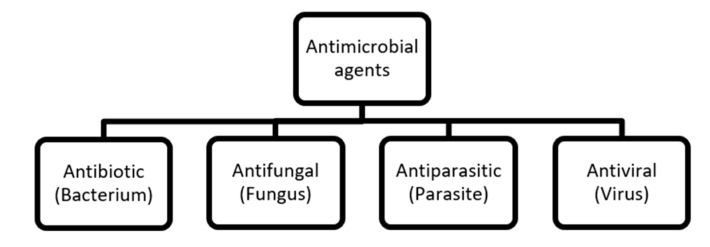
Classification of drug-based antimicrobial agents [11].

The group of biocides includes a great variety of substances which can be classified according to their mechanism of biocidal action [8]. A few classifications have been proposed. Chapman (2003) has suggested that biocides can be divided into two main groups: electrophiles and membrane active biocides [8]. In the electrophile group, typical electrophiles and oxidants are distinguished. The mechanism of action of oxidants is based on the reaction using radical transfer. Halides and peroxides belong to this biocide group. The electrophile group owes its biocidal properties to covalent interactions aimed at inactivating enzymes. This creates intracellular free radicals and consequently, cell death. On the other hand, membrane active biocides are the compounds that show activity directed to cells’ membranes. These include biocides with lytic activity and protonophores. Lytic biocides have a mechanism of action based on the destabilization of the cell membrane structure. This action breaks the cell membrane and leads to rapid cell lysis. Such a mechanism of biocidal activity is demonstrated, among others, by phenols, quaternary ammonium salts (QASs), as well as some alcohols, e.g., phenoxyethanol. Protonophore biocides interfere with the ability of the cell membrane to maintain an appropriate pH balance. The strong acidification of the interior of the cell, which is a consequence of the application of this group of biocides, disrupts the cellular metabolism. The compounds showing this mechanism of action include parabens and sorbic acid and benzoic [8,9,10]. Lakshmi et al. (2018) have suggested that the agents which kill or impede the growth of microorganisms are antimicrobials [11]. The classification proposed by these authors is presented in Figure 1. 

Denyer et al. have indicated that the vegetative bacterial cell offers three broad regions to biocide interaction: the cell wall, cytoplasmic membrane and the cytoplasm. The biocide access to these regions is determined by the extracellular material, cell morphology and the cellular chemical composition [12,13,14]. According to the mechanisms of antibacterial action, these agents are now divided into the following five main groups whose mechanisms are [13,15,16,17,18]:

- Inhibition of cell wall synthesis;
- Inhibition of protein synthesis;
- Inhibition of nucleic acid synthesis;
- Inhibition of metabolic pathways;
- Interference with cell membrane integrity.

McDonnell and Russell (1999) have analyzed the mechanisms of action of biocides and emphasized both the interaction at the cell surface and intracellular action of biocides, which can produce a significant effect on viability. The outermost layers of microbial cells can thus have a significant effect on the cells’ susceptibility (or insusceptibility) to antiseptics and disinfectants. The same authors have indicated several possible mechanisms of action of biocides such as: the cross-linking of proteins; the generalized membrane damage involving phospholipid bilayers; the cross-linking of RNA and DNA; the intercalation of an acridine molecule between two layers of base pairs in DNA; membrane-bound enzymes (interaction with thiol groups); the inhibition of DNA synthesis; and the oxidation of thiol groups to disulfides, sulfoxides, or disulfoxides [19]. There are generally five mechanisms of antimicrobial agents’ activity against fungus cells, based on the inhibition of microbial cell wall synthesis, DNA synthesis function, protein synthesis, folic acid metabolism and the destruction of membrane [11].

The cell wall structure of Gram-positive and Gram-negative bacteria is different. The difference is that the Gram-negative bacteria have an outer membrane of the cell wall, made of proteins, phospholipids and lipopolysaccharide. This structure generally limits the penetration of various biocides as well as amphiphilic compounds into the cell [20,21]. The differences in the cell wall structure determined the antimicrobial activity of the biocides. It has been established that this activity is varied and depends on both the biocide structure and the type of microorganism it is interacting with.

## 2. Biocidal Action of Quaternary Ammonium Salts

**Figure 2:**
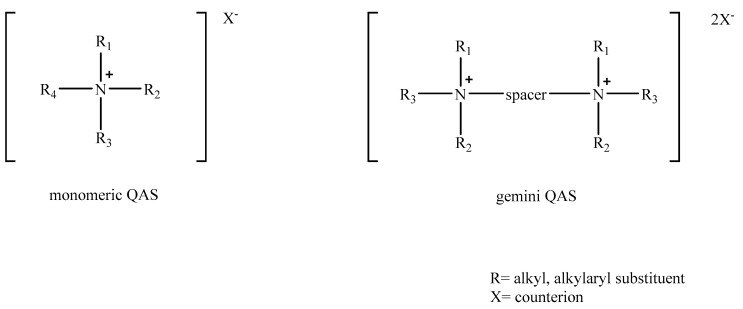
General formulas of the aliphatic quaternary ammonium salts (QAS) with monomeric and the gemini structure.

Analyzing the world surfactant market, it can be summarized that cationic surfactants have found applications as fabric softeners, asphalt additives, corrosion inhibitors and textile auxiliaries and biocides. Taking into account this wide range of their applications, it is not surprising that cationic surfactants account for almost 7% of the total surfactant market [22]. The whole group of cationic surfactants have a characteristic structure with a positively charged hydrophilic head and a hydrophobic tail usually made of a long alkyl chain in one molecule. Taking into account the difference in the structure of the hydrophilic head, the cationic surfactants were divided into four groups: amines, quaternary ammonium salts, sulfonium salts and phosphonium salts. In the 1930s, Domagk described the antimicrobial properties of quaternary ammonium salts. Since then, several generations of these surfactants have been created, resulting in a group of quaternary ammonium surfactants, being a diversified group in terms of chemical structure. The most general division of this class of surfactants assumes the distinction of derivatives with monomeric, dimeric (gemini), trimeric and polymeric structures. The following is the general formula for aliphatic monomeric and gemini quaternary ammonium salts (Figure 2).

The central nitrogen atom of the aliphatic salt is substituted with alkyl or alkylaryl groups and due to the type of substituent, the salts are: alkylammonium, alkylmethylammonium, alkyl dimethylammonium, alkyltrimethylammonium, alkylbenzyldimethylammonium, dialkyldimethylammonium, trialkylmethylammonium. In the group of compounds classified as quaternary ammonium salts, in addition to aliphatic salts, one can find a large group of salts that are heterocyclic derivatives, e.g., pyridinium, imidazolinium, imidazolium, benzimidazolium, quinoline, isoquinoline, piperidinium, morpholinium, benzamidinium. 

### 2.1. Biocidal Action of Monomeric Quaternary Ammonium Salts

#### 2.1.1. Antibacterial Activity of Quaternary Ammonium Salts

Quaternary ammonium salts have a broad spectrum of biological activity, showing among others, the following effects: algistatic, bacteriostatic, tuberculostatic, sporostatic, and fungistatic. Their activity against viruses is also known [23,24,25,26]. 

According to the literature, despite numerous studies over many years, the mechanism of antimicrobial activity has not been fully understood [27]. As reviewed by Tischer et al., the mechanism of action of QASs can be summarized as follows. At the first stage, the QAS molecule adsorbs on the cell wall and penetrates it [28]. Further activity assumes the reaction with lipids and proteins of the cell membrane, which leads to disorganization in its structure and the leakage of low-molecular components out of the cell. Then, proteins and nucleic acids degrade inside the cell. The release of autolytic enzymes leads to the lysis of the cell wall components. The observable effect of these processes is a complete loss of the structural organization of the cell [28]. In more detailed molecular terms, the mechanism of QAS action involves the association of a positively charged quaternary nitrogen with phospholipid acids in the membrane. Then, the hydrophobic tail penetrates the hydrophobic membrane core. Exposure to QAS causes, as a consequence, an increase in the surface pressure and the membrane transformation from a liquid to a liquid crystal state. Osmoregulatory and physiological functions of the membrane are also lost. The membrane core hydrophobicity is reduced and the phospholipids that are the basic building blocks of the membrane tend to form hexagonal systems. This course of biological activity of QAS occurs when the biocide concentration is approximately equal to the minimum inhibitory concentration (MIC). At higher concentrations, aggregates are formed that solubilize hydrophobic membrane elements [29]. It has also been shown that membrane activity is related to the length of the alkyl chain as well as the size of the surfactant polar head [23]. Usually, the highest biocidal activity shows QASs with 10–12 carbon chains, while the extension and the reduction in the length of the alkyl chain weakens the antimicrobial activity [23]. Opinions on this issue are divided, and the available literature indicates that the highest biocidal activity towards Gram-positive bacteria and yeasts exhibit QASs with 12–14 carbon chains, while QASs with 14–16 carbon chains are biocidal against Gram-negative bacteria [30]. The biocidal activity of QASs is different towards Gram-positive and Gram-negative bacteria. A biocidal agent is only active when it can pass through the outer layers of the cell, whose structure and composition make it act as a barrier [31]. Knowing this, it is not surprising that Gram-negative bacteria more often show resistance [31]. 

#### 2.1.2. Bacterial Resistance to Quaternary Ammonium Salts

In the struggle for survival, bacteria also implement the mechanism of acquired and internal resistance. Internal resistance may be developed through the following mechanisms: impermeability, efflux and inactivation. In turn, acquired resistance can be manifested in several ways, but the most important are mutations or the acquisition of genetic material in the form of plasmids or transposons. Furthermore, acquired resistance may be developed through the mechanisms of inactivation/modification, insensitive target sites, by-pass of sensitive steps, the overproduction of targets or the absence of an enzyme metabolic pathway [32,33].

When considering the resistance, the phenomena of cross- and co-resistance must also be taken into account. When different biocides attack the same microorganism, initiating a common path leading to cell death, or sharing a common route of access to their respective targets, then cross-resistance may occur. If the genes that determine resistant phenotypes coexist on e.g., plasmid, transposon or integron, then co-resistance may occur. It is manifested by the development of resistance to one biocide by the emergence of resistance to another antimicrobial agent [8]. The growing resistance of microorganisms to antibiotics and the widespread use of QASs in healthcare, observed in recent years, prompts reflection on the cross-resistance of antibiotics and QASs. One of the main mechanisms of resistance to antibiotics and biocides is the mechanism of active efflux [31]. Efflux systems with broad specificity are observed in all bacteria. Their aim is to pump the disinfectant out of the cell and thereby reduce its concentration inside. For *Staphylococcus aureus*, six qas genes (qasA, B, C, G, H, J) have been described among the plasmid-encoded MDR (multidrug resistance) efflux pumps. The qasA and qasB genes are usually encoded on large plasmids, while the QAS resistance genes qasG, qasH, and qasJ are found on small plasmids. The qasA protein is the most common pump mediating the resistance to QASs. The qasC gene, which is responsible for coding a membrane efflux protein from the small multidrug resistance (SMR) family, is detected slightly less frequently. The qasB protein is responsible for reducing sensitivity to diamidines and biguanides [31,34]. 

The relationship between biocide- and antibiotic-resistant genes and the mobile genetic elements has been confirmed. Already in the late 1990s, it was found that in *Staphylococcus aureus*, as a qasA gene, is transferred onto plasmid pSK01, it also carries genes responsible for resistance to trimethoprim (drfA) and aminoglycosides (aacA-aacD) [35]. The qasA/B genes were detected in clinical isolates. The same plasmids contained also β-lactamases and heavy metal resistance determinants. For the Gram-negative bacteria, the presence of two genes—*Enterobacteriaceae* and *Pseudomonas spp*.—in one gene cassette, that encoded quaternary ammonium and sulfonamide resistance, respectively, was found [36,37].

#### 2.1.3. Antifungal Activity of Quaternary Ammonium Salts

QASs also exhibit biological activity against filamentous fungi and yeast, however, this activity cannot be described by the same mechanism as that against bacteria. Studies on hexadecyltrimethylammonium bromide (CTAB) activity indicate that a key process in the phenomenon of antifungal activity is the reversal of the distribution of charges on the cell surface. It is assumed that the negative charge is replaced by a positive one, whilst simultaneously, the cell membrane is not disturbed, a phenomenon that facilitates the penetration of the QAS molecule through the cell wall [38]. In recent years, biocidal and inhibitory properties against filamentous fungi and yeasts including *Aspergillus ochraceus*, *Candida krusei*, *Candida parapsilosis*, *Aspergillus flavus*, *Aspergillus niger*, *Fusarium spp.*, *Cladosporium spp.* have been widely studied [39]. In addition, auxotrophic yeast mutants and mutants with respiratory defects of nuclear (pet) and mitochondrial (rho^-^, rho^0^) genes have been found to be sensitive to QASs [40]. 

### 2.2. Biocidal Action of Gemini Quaternary Ammonium Salts

Gemini surfactants are derivatives of QAS that exhibit a broader biocidal activity spectrum than monomeric QASs. Koziróg and Brycki have compared dodecyltrimethylammonium bromide (DTAB) and its gemini analogue and have shown that the MIC value of the gemini derivative was 70 times smaller than that of *Staphylococcus aureus*. This trend has also been confirmed for other common pathogens, e.g., *Pseudomonas aeruginosa*, *Candida albicans* [41]. In addition to their excellent biocidal properties, gemini surfactants also exhibit better surface properties than monomeric QASs [42]. It is estimated that gemini surfactants show better wetting properties and their biodegradability is comparable to that of monomeric QASs. Although extensive research has been conducted for years on the properties of gemini quaternary surfactants, the mechanism of their biocidal activity has not been fully understood [43]. This is probably due to the huge variety of chemical structures in this class of surfactants. It has been evidenced that gemini QASs having 10–12 carbon chains show the highest biocidal activity against bacteria and microscopic fungi [44]. The length and nature of a spacer are also significant in this activity. In addition, bromides have a higher biocidal activity than chlorides of analogous chemical structure [44]. For the QAS gemini derived from pyridine, Sumitomo and colleagues have proposed a three-stage biocidal mechanism of action [45]. The first stage involves the displacement of Mg^2+^ ions from the outer layer of the cell membrane and their replacement with surfactant particles. This leads to the formation of bulges on the surface of the cell membrane due to the fact that the size of the surfactant molecule is much larger than that of the Mg^2+^ cation. The second stage is the inhibition of respiratory enzyme activity and the leakage of cell membrane components such as lipopolysaccharides (LPS) and outer membrane pore protein E (OmpE). The formation of bubbles and bulges on the surface of the cell membrane as well as the destruction of peptidoglycans causes permanent irreversible damage to the membrane structure and the leakage of internal cellular components. This is the third final stage of the biocidal activity of gemini QASs [45].

#### 2.2.1. Activity of Gemini Quaternary Ammonium Salts Against Bacterial Biofilm Formation

In recent years, much effort has been devoted to the problem of fighting against biofilm formation by bacteria. The literature defines the concept of biofilm as closed in the matrix populations of bacteria adherent to each other and / or to surfaces or interfaces [46]. The formation of biofilm in a hospital environment is particularly dangerous because bacterial cells entering the biofilm show increased resistance to biocides, in addition, they have a changed metabolism [43]. One of the bacteria that is associated with the formation of biofilm on medical devices is *P. aeruginosa* [43]. These bacteria may be responsible for urinary tract infections, peritonitis and, in patients with cystic fibrosis, for lung infections. Literature provides evidence clearly indicating that surfactants classified as gemini QAS can be used as active compounds in biocidal preparations against the biofilm created by *P. aeruginosa* [46]. A possible mechanism of gemini surfactant activity against biofilm assumes the occurrence of electrostatic interactions that disrupt its functioning, and as a consequence, biofilm cell lysis [47]. In the case of biofilm formed by Gram-positive bacteria, gemini QASs also show biofilm-eliminating activity, which has been confirmed e.g., for alanine gemini surfactants that manifested antibiofilm activity against *Staphylococcus epidermidis* [40]. However, the research efforts have not been directed only at finding a biocide that would permanently destroy the biofilm, it is also considered essential to prevent microbial adhesion. For this reason, numerous studies have been devoted to the creation of surfaces modified with QASs and their gemini derivatives. It is expected that the surfaces prepared in this way will help to control the phenomenon of adhesion of microorganisms [40]. 

#### 2.2.2. Antifungal Activity of Gemini Quaternary Ammonium Salts

Gemini QAS derivatives, similarly to monomeric QASs, apart from their biocidal and inhibitory activity against bacteria, also show activity against yeast and filamentous fungi. Shirai and co-workers have proposed a mechanism of the biocidal effect of gemini QASs on the baker’s yeast *Saccharomyces cerevisiae*. They tested 3,3’- (2,7-dioxaacetane) bis (1-decylpyridinium bromide) gemini surfactant substituted with two 10-carbon chains. The authors have found that the tested compound does not destroy the cell wall but penetrates it and leads to the breaking of the membranes of internal organelles [48]. 

The interaction of *C. albicans* and gemini QAS is particularly interesting and important because this human fungal pathogen has shown strong resistance to drugs used until now [49]. Obłąk and co-workers have reported that the gemini compounds studied by them showed biocidal activity at the same time against *C. albicans* and *Rodotorula mucilaginosa*. Morevoer, the ability to eradicate *C. albicans* and *R. mucilaginosa* biofilm was observed. The most important observation resulting from these studies was that the gemini surfactants tested increased the sensitivity of *C. albicans* to azoles and polyenes. This gives hope for the possibility of using combination therapies against drug-resistant *C. albicans* [49]. 

## 3. Biocidal Action of Zwitterionic Surfactants

Zwitterionic surfactants are electroneutral salts possessing, in the same molecule, two ionic centers of different charge. For most zwitterionic surfactants, the cationic moiety consists of a cationic quaternary ammonium group, while the anionic moiety includes a carboxylic acid, sulfonic acid, sulfuric acid ester, or phosphoric acid ester [50,51]. Zwitterionic surfactants include sulfobetaines. The positively charged moiety of sulfobetaines is the quaternary nitrogen atom, while the negatively charged moiety is the sulfonate group. The hydrophobic part of the molecule is the alkyl chain. Therefore, sulfobetaines are internal, electroneutral salts in which the hydrophilic cationic and anionic groups coexist in the same molecule [50,51]. Sulfobetaines can be used as disinfectants, although their effectiveness towards microorganisms is diverse. Sulfobetaines, whose antimicrobial properties have been widely presented in literature, show high antibacterial activity against Gram-positive and Gram-negative bacteria with an indication of higher activity against the former ones [52]. According to the available literature, Gram-negative bacteria are often more resistant to various biocides than Gram-positive ones [52]. Many authors have emphasized the possibility of using sulfobetaine derivatives to prevent biofilm formation by bacteria such as *S. epidermidis*, *Pseudomonas putida* and *P. aeruginosa* [53,54,55]. It has been shown that the antimicrobial activity of the sulfobetaines studied is varied and depends on both their structure and the type of microorganism they are interacting with. It has been established that sulfobetaines with 10 carbon atoms in the alkyl chain do not show antibacterial activity against indicator strains, while their homologues with 12, 14 and 16 carbon atoms show a broad spectrum of activity [56,57]. Among the Gram-negative bacteria tested, *Aeromonas hydrophila*, *Escherichia coli*, *Proteus vulgaris and Pseudomonas aeruginosa*, *E. coli* was the most resistant to the compounds tested, whereas *P. aeruginosa* and *A. hydrophila* were the most sensitive to them. The growth of all the tested Gram-positive bacteria was inhibited by sulfobetaines, however, their sensitivity varied. The strongest inhibition was observed for *S. aureus*, *Bacillus subtilis* and *Bacillus megaterium,* treated with sulfobetaines with 12, 14 and 16 carbon atoms [56,57].

The latest literature data show that the studies on sulfobetaine application concentrate on the synthesis of polymeric compounds with zwitterionic moieties. Sulfobetaine polymer-based materials exhibit highly ionic character with balanced charges between the sulfate and quaternary ammonium group in each monomer unit and enter into strong electrostatic interactions with water [58]. These zwitterionic polymers bear an equimolar number of homogenously distributed anionic and cationic groups along their polymer chains. This combination of oppositely charged moieties grants the polymers their ultra-hydrophilicity, while, at the same time, maintaining an overall charge neutrality [59]. Zwitterionic materials, including phosphobetaine, sulfobetaine and carboxylbetaine, have become the new generation of robust fouling resistant biomaterial systems beyond PEG (Polyethylene Glycol). Widely used sulfobetaine methacrylate (SBMA), which can form an ultra-hydrophilic interface, has been approved for its general antifouling performance, including resistance to non-specific protein adsorption, blood cell attachment, human tissue adhesion, and bacteria attachment [60]. Zwitterionic polymers have a broader chemical diversity and greater freedom for molecular design. The microbiological applications of zwitterionic polymers and their derivatives must be emphasized, as well as the integration of antimicrobial and nonfouling properties. Zwitterionic polymer-based materials naturally create a region of high osmotic pressure at the interface. According to Mi and Jiang (2014), who studied PEG polymers as model systems, the osmotic pressure exerted by a polymeric scaffold has a profound impact on bacterial physiology and gene expression profile, including the production of virulent factors [59]. The latest research has shown that sulfobetaine polymers can be used for wound dressing [61,62] or as the interface material in medical device coating [60]. In general, sulfobetaine copolymers show good antibacterial activity against some Gram-negative and Gram-positive bacteria and the surface covered with them is resistant to bacterial adhesion and biofilm formation [60].

## 4. Current and Future QAS Application Trends

The research reports that have appeared over the last several months clearly show that the interest of researchers is to a great extent focused on the medical use of QAS biocidal properties. Due to the threat arising from hospital-acquired infections, the subject of the correlation between the presence of antiseptic resistance genes and the resistance to various antibiotics is discussed in the literature. The authors emphasize the need for further research into the importance of the presence of genes controlling resistance to antiseptics [63]. This need is confirmed in Han and co-workers’ research, in which they prove that QACs may facilitate the evolution and gene transfer of antibiotic resistance gene among microbiome [64].

The results of studies on the use of QASs in dental materials are promising. Much effort is directed to develop dental composites and binding agents modified with QASs that will limit the development of oral bacteria that cause tooth decay and inhibit the growth of their biofilm [65,66]. In addition, ionomer glass cements are currently modified with QAS, in order to improve their performance [67]. An interesting potential medical application may be the dressings based on bacterial cellulose and QASs. Preliminary studies have shown the possibility of obtaining a wound dressing that would simultaneously exhibit antimicrobial activity and biocompatibility [68]. An interesting and promising solution is also the use of QAS-modified polyurethane foams for the production of dressings. Tran and co-workers proved that such a dressing inhibited the growth and development of bacterial biofilm, and also inhibited bacterial migration [69]. Surgical sutures coated with QASs may also have a beneficial effect on the inhibition of postoperative infections [70]. The problem for the healthcare system is also the occurrence of fungal infections caused by drug-resistant *C. albicans*, gemini quaternary ammonium salts may be helpful in fighting them [71]. Available literature also indicates the possibility of using antimicrobial composites based on cellulose nanocrystals and QAS as coating agents in antiseptic packaging [72]. 

Apart from the applications correlated with medicine, the prospects for using quaternary ammonium salts in the technology of water and wastewater treatment are visible [73]. The frequency of using membrane technologies in these processes is increasing, but the limiting factor is the adhesion of microorganisms followed by biofilm formation. The solution to this problem may be membrane formation and the grafting of quaternary ammonium salts on a silica-decorated surface. Zhang et al. saw this method as a way to modify a wide range of membranes to reduce biofouling [73]. 

Surface modification with quaternary ammonium salt molecules remains a common topic undertaken by scientists around the world. Not without significance, is the constant search for new derivatives of quaternary ammonium salts that would additionally have biocidal potential. Research conducted in recent years has made it possible to obtain branched tetracationic derivatives of quaternary ammonium salts (multiQAS) having activity against methicillin-resistant Staphylococcus aureus [74].

The efforts for obtaining new zwitterionic surfactants of sulfobetaine structure that would exhibit antibacterial properties are continued [75]. Recently, zwitterionic surfactants have found interesting application as a component of a multifunctional thin-film nanofiber composite membrane, which showed excellent properties: anti-biofouling (e.g., hydrophilicity, lower bovine serum albumin (BSA) adsorption, higher-water flux recovery and less *E. coli* and *S. aureus* bacteria attachment) and blood compatibility [76]. These properties make it possible to use such a composite in biomedical engineering in future [76]. 

It seems that continuing research on obtaining new materials with antibacterial properties is justified. Unfortunately, the literature is poor in the detailed data on the mechanisms of biocidal activity directed against yeasts and filamentous fungi. Nevertheless, attempts at providing information on the methods of synthesis of new surfactants and their surface activity are continued.
